# Structural Identifiability and Observability of Microbial Community Models

**DOI:** 10.3390/bioengineering10040483

**Published:** 2023-04-17

**Authors:** Sandra Díaz-Seoane, Elena Sellán, Alejandro F. Villaverde

**Affiliations:** 1 Department of Systems Engineering & Control, Universidade de Vigo, 36310 Vigo, Spain; sandra.diaz@uvigo.gal (S.D.-S.); sellanelena@gmail.com (E.S.); 2 CITMAga, 15782 Santiago de Compostela, Spain

**Keywords:** dynamic modelling, systems biology, identifiability, observability, microbial communities

## Abstract

Biological communities are populations of various species interacting in a common location. Microbial communities, which are formed by microorganisms, are ubiquitous in nature and are increasingly used in biotechnological and biomedical applications. They are nonlinear systems whose dynamics can be accurately described by models of ordinary differential equations (ODEs). A number of ODE models have been proposed to describe microbial communities. However, the structural identifiability and observability of most of them—that is, the theoretical possibility of inferring their parameters and internal states by observing their output—have not been determined yet. It is important to establish whether a model possesses these properties, because, in their absence, the ability of a model to make reliable predictions may be compromised. Hence, in this paper, we analyse these properties for the main families of microbial community models. We consider several dimensions and measurements; overall, we analyse more than a hundred different configurations. We find that some of them are fully identifiable and observable, but a number of cases are structurally unidentifiable and/or unobservable under typical experimental conditions. Our results help in deciding which modelling frameworks may be used for a given purpose in this emerging area, and which ones should be avoided.

## 1. Introduction

Computational systems biology relies heavily on dynamic modelling to understand the mechanisms of complex biological processes, with the ultimate goal of controlling and optimising them [1]. To this end, control-theoretic concepts such as structural identifiability and observability are being increasingly applied in this research area. Observability is the possibility of inferring the system’s internal state from knowledge of its input and output [2], while identifiability is the possibility of inferring its parameters [3]. Since parameters can be considered as constant states, identifiability can be seen as a particular case of observability. We refer to the joint analysis of structural identifiability and observability as ‘SIO’. The concept of observability was introduced by R.E. Kalman in the 1960s and was later extended to nonlinear systems in the 1970s [4]. Since the analysis of SIO can be very challenging in nonlinear models, its application in biological modelling has become widespread only recently, thanks to computational advances [5].

Microbial communities or consortia are a specific class of biological systems that result from the coexistence of different species of microorganisms. Despite their abundance, detailed information on the composition and function of microbial communities (both within humans and Earth-wide) has been obtained only in the last few decades [6], thanks to experimental advances in molecular biology. Likewise, the application of control-theoretic techniques to their analysis is largely unexplored, despite a number of recent examples [7,8,9]. The development of accurate and informative mathematical models is a key tool for understanding, predicting, and controlling the behaviour of microbial consortia [10,11]. Modelling efforts often focus on bacteria, but some models also include bacteriophages, which are viruses (also called phages) that infect bacteria [12]. Nonlinear systems of ordinary differential equations can be used to describe the different types of interactions present in these communities and their emergent dynamics. Different types of models are used to this end. In some of them, such as the generalized Lotka–Volterra (gLV) models, the dynamics arise from species–species interactions, which may be competitive or cooperative. Another approach, that of species–metabolite interaction models, describes the emergent behaviour of the community as a result of the competition for common resources. Several versions of these models have been presented in the literature [13,14,15,16].

The parameters in microbial community models may have different meanings, such as quantifying the degree of cooperation or competition between species, or representing growth, degradation, or dilution rates. Therefore, knowledge of their values can provide valuable insight about community dynamics. However, our ability to build informative models can be greatly influenced by experimental limitations. Species abundances are often inferred solely from high-throughput DNA sequence data. The resulting datasets provide estimates of relative abundances, but typical modelling approaches—including gLV—describe absolute abundances instead. Remien and coworkers [17] analysed the structural identifiability of gLV models with absolute and relative data, as well as the local practical identifiability of a synthetic community. Interestingly, they found that certain interaction parameters were unidentifiable from relative data.

As structural identifiability is a requirement for successful parameter estimation, observability is a requirement for successful state estimation. Since non-identifiability and non-observability are often linked (e.g., due to one or more parameters being correlated with a given state variable), both properties (SIO) need to be analysed jointly. The lack of SIO can sometimes, but not always, be remedied by modifying the experimental setup so as to measure more state variables or functions of them. Even when it is possible to first perform parameter estimation offline, and then use the parameter estimates for the online estimation of the unknown state variables using a state observer, it is crucial to ensure the SIO of both experimental setups (online and offline). Otherwise, errors in the estimates of the unknown parameters would be propagated to the estimates of the unmeasured states. Since the SIO of a model determines the possibility of inferring its parameters and internal state, it is crucial to analyse these properties to guarantee the reliability of the modelling results. Thus, systematic studies on the SIO of the models available for a particular application are useful resources; see, e.g., [18]. However, such analyses of the properties of the most widely used microbial community models are currently lacking. In the present paper, we perform the said study. We analyse three main types of models: species–species interaction models, which include the aforementioned gLV as well as a variant of them devised for compositional data; species–metabolite interaction models, including quadratic interactions and enzymatic kinetics, and a third class of models that are suited for therapeutic applications with phage cocktails.

## 2. Materials and Methods

### 2.1. Definitions

We study models that assume that the populations of each microorganism are sufficiently large to be well described by deterministic equations. Furthermore, we neglect spatial variability. Therefore, we use ordinary differential equation (ODE) models that can be written as

(1) $$M:\left\{\begin{array}{c} \begin{array}{ccc} \dot{x}\left(t\right) & = & f(x(t),u(t),\theta ),\\ y(t) & = & h(x(t),u(t),\theta ). \end{array} \end{array}\right.$$

where $x\left(t\right)\in R^{n}$, $u\left(t\right)\in R^{q}$, $\theta \in R^{p}$, and $y\left(t\right)\in R^{m}$ represent the state, input, parameter, and output vectors, respectively. We refer to their elements with subindices, e.g., $\theta_{i}$, $x_{j}$. The inputs are assumed to be known, while the parameters are, in principle, unknown. The outputs are the measured quantities, which often consist of direct measurements of state variables, although they may also be functions of them.

Broadly speaking, we say that a model of the form (1) is *observable* (respectively, *structurally identifiable*) if its state vector x(t) (respectively, parameter vector θ) can be determined by measuring its future outputs y(t) and inputs u(t) in a bounded time horizon. We consider *local* versions of these properties, which hold in a neighbourhood of each variable.

More formally, we say that a parameter $\theta_{i}$ of (1) is *structurally locally identifiable* (SLI) if, for almost all vectors $\theta^{*}$, there is a neighbourhood $N(\theta^{*})$ in which the following condition holds:

(2) $$\hat{\theta }\in N\left(\theta^{*}\right)\;\mathrm{and}\;y\left(t,\hat{\theta }\right)=y\left(t,\theta^{*}\right)\Rightarrow \hat{\theta_{i}}=\theta_{i}^{*}$$

If (2) is not fulfilled, $\theta_{i}$ is *structurally unidentifiable* (SU). If all its parameters are SLI, the model is SLI; otherwise, it is SU.

Similarly, a state variable $x_{i}\left(\tau \right)$ is *observable* if it can be distinguished from any other neighbouring states from knowledge of the output y(t) and input u(t) vectors in the interval $t_{0}\leq \tau \leq t\leq t_{f}$, where $t_{f}$ is finite. Otherwise, $x_{i}\left(\tau \right)$ is *unobservable*. A model is called observable if all its states are observable; otherwise, it is unobservable.

We use the acronym FISPO (full input, state, and parameter observability) to refer to a model that is SLI and observable [19].

### 2.2. Analysis Methods

The structural local identifiability and observability (SIO) of a nonlinear model can be determined with a differential geometry approach, applying techniques based on the concepts presented, e.g., in [4]. To this end, the state variables and parameters are included in an augmented state vector, $\tilde{x}=\left[x,\theta \right],$ and an observability–identifiability matrix $O_{I}^{NL}\left(\tilde{x}\right)$ is calculated as follows [20]:

(3) $$\begin{array}{c} O_{I}^{NL}\left(\tilde{x}\right)=\left(\begin{array}{c} \frac{\partial }{\partial \tilde{x}}h\left(\tilde{x}\right)\\ \frac{\partial }{\partial \tilde{x}}\left(L_{f}h\left(\tilde{x}\right)\right)\\ \frac{\partial }{\partial \tilde{x}}\left(L_{f}^{2}h\left(\tilde{x}\right)\right)\\ \vdots \\ \frac{\partial }{\partial \tilde{x}}\left(L_{f}^{n+p-1}h\left(\tilde{x}\right)\right) \end{array}\right) \end{array}$$

where $L_{f}h\left(\tilde{x}\right)$ is the Lie derivative of the output,

(4) $$L_{f}h\left(\tilde{x}\right)=\frac{\partial h(\tilde{x})}{\partial \tilde{x}}f\left(\tilde{x},u\right)+\sum_{j=0}^{j=\infty }\frac{\partial h(\tilde{x})}{\partial u^{\left(j\right)}}u^{\left(j+1\right)}.$$

and higher-order derivatives can be obtained as

(5) $$L_{f}^{i}h\left(\tilde{x}\right)=\frac{\partial L_{f}^{i-1}h\left(\tilde{x}\right)}{\partial \tilde{x}}f\left(\tilde{x},u\right)+\sum_{j=0}^{j=\infty }\frac{\partial L_{f}^{i-1}h\left(\tilde{x}\right)}{\partial u^{\left(j\right)}}u^{\left(j+1\right)}$$

If $O_{I}^{NL}\left(\tilde{x}\right)$ has full rank, i.e., n+p, the model is FISPO. Note that the *i*th column of $O_{I}^{NL}\left(\tilde{x}\right)$ contains partial derivatives with respect to the *i*th element of $\tilde{x}$, which is either a parameter or a state variable. Therefore, when $\mathrm{rank}(O_{I}^{NL}\left(\tilde{x}\right))<n+p$, the identifiability of each parameter and the observability of each state variable can be determined by removing the corresponding column and recalculating the matrix rank [2]: if the rank decreases, the corresponding parameter (respectively, state variable) is SLI (respectively, observable). If it remains constant, the parameter is SU (respectively, the state variable is nonobservable).

There is an alternative method that calculates the matrix rank more efficiently. It adopts a differential algebra approach [21] and allows us to compute the set of observable variables in polynomial time. For the purpose of notation, capital letters are used for the initial conditions of a function and its derivatives, i.e., $u^{\left(r\right)}\left(0\right)=U^{\left(r\right)}$ and $y^{\left(r\right)}\left(0\right)=Y^{\left(r\right)}$ for r≥0 and then $U=(U^{\left(0\right)},U^{\left(1\right)},...)$, $Y=(Y^{\left(0\right)},Y^{\left(1\right)},...)$. Moreover, having $U_{i}^{\left(0\right)},U_{i}^{\left(1\right)},...$ for $i=1,...,n_{u}$ and $Y_{j}^{\left(0\right)},Y_{j}^{\left(1\right)},...$ for $j=1,...,n_{y}$, then $R\langle U,Y\rangle$ denotes the field adjoining the indeterminates to R. In [21], the transcendence degree is also calculated with the rank of $O_{I}^{NL}\left(\tilde{x}\right)$. In order to compute the matrix, a different approach is taken to avoid the expensive calculation of the Lie derivatives. The underlying procedure can be seen in Algorithm 1.

We have implemented both methods in the Matlab toolbox STRIKE-GOLDD [22]. In the analyses reported in this paper, we have used preferentially the second, most efficient algorithm.

Finally, we note that it is possible to extend the approach defined in this section to models with unmeasured inputs; see [19,23,24] for details.
**Algorithm 1:** Probabilistic algorithm to test local algebraic observability in polynomial time**Preprocesing** Construct a straight-line program encoding the variational system ∇P with $P=\dot{\tilde{x}}-f\left(u\left(t\right),\tilde{x}\left(t\right)\right)$ and the expressions used during its integration. **Specialization** Specialisation of the parameters, $\theta^{*}$, and the inputs, $u^{*}$ Power Series Solution Computation of the power series solution of ∇P at order $n_{\tilde{x}}+1$ with a specialised value for the states **Jacobian computation** Evaluation of ∇y on the previous results, giving the coefficients of the Jacobian matrix **Rank computation** Calculation of the matrix rank and transcendence degree **if**
*transcendence degree = 0*
**then** |    System is algebraically observable **else** |    Determine which variable or variables are not observable. **end**

## 3. Models

**Figure 1:**
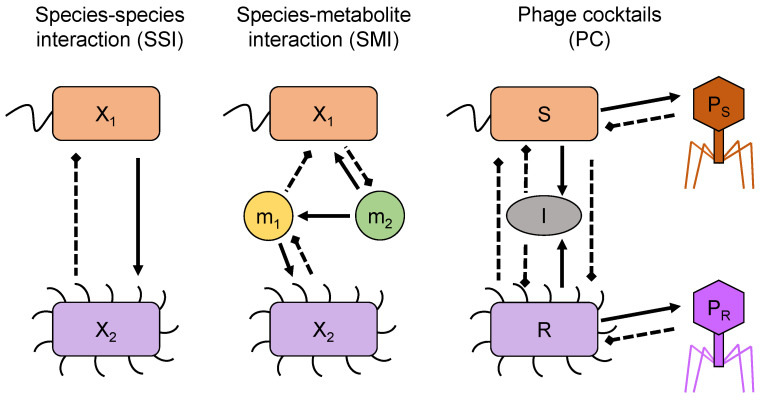
The three main classes of microbial community models analysed in this paper. Positive interactions (i.e., which increase the abundance of the entity to which they point) are represented by solid arrows, while negative interactions (which decrease the abundance of the target) are represented by dashed lines with a square end. SSI models (left) describe the dynamics as direct competition or cooperation between species (depicted as two different bacteria). SMI models (central panel) describe the dynamics as the result of competition for resources. In the diagram, bacterium $X_{1}$ consumes metabolite $m_{2}$, while $X_{2}$ consumes $m_{1}$; furthermore, $m_{2}$ can also be transformed into $m_{1}$, which is a by-product detrimental to $X_{1}$. The panel on the right depicts a phage cocktail (PC) in which each phage ($P_{S}$, $P_{R}$) attacks a specific bacterium; the immune response *I* is also included.

A number of microbial community models have been proposed within the framework defined by (1); we describe them in the remainder of this section. Their diagrams are shown in Figure 1.

### 3.1. Species–Species Interaction Models (SSI)

Species–species interaction models (SSI) assume that the dynamic behaviour can be modelled as the result of direct interactions between species. If we only take into account two-way interactions between species, the dynamics of each species is given by

(6) $${\dot{x}}_{i}=h_{i}\left(x_{i}\right)+\sum_{j=1}^{n}f_{ij}\left(x_{i},x_{j}\right)$$

where $x_{i}$ is the abundance of species *i*, $h_{i}$ its intrinsic growth, and $f_{ij}$ describes the increase or decrease in species *i* due to the interaction with species *j*. Typical SSI models include the classic Lotka–Volterra models of predator–prey interactions from community ecology, as well as different versions of them.

#### 3.1.1. Generalized Lotka–Volterra Models (gLV)

The generalized Lotka–Volterra model (gLV) is of the form (6), with the assumption that $h_{i}=r_{i}\cdot x_{i}$ and $f_{ij}=\beta_{ij}\cdot x_{i}\cdot x_{j}$. This yields

(7) $${\dot{x}}_{i}=r_{i}\cdot x_{i}+\sum_{j=1}^{n}\beta_{ij}\cdot x_{i}\cdot x_{j}$$

Thus, gLV models have one $r_{i}$ parameter per state variable, which represents its growth rate, and one $\beta_{ij}$ parameter for each pair of state variables, which represents their interaction rate. Note that the latter are not necessarily symmetrical, i.e., $\beta_{ij}\neq \beta_{ji}.$

#### 3.1.2. Composite Lotka–Volterra Models (cLV)

The composite Lotka–Volterra framework (cLV) was introduced by [14]. It was motivated by the fact that microbiome datasets are often obtained by high-throughput sequencing and are therefore *compositional*, i.e., they have an arbitrary total imposed by the instrument [25]. In many practical applications, only relative abundance measurements are available; however, the state variables in gLV models represent absolute abundances. By applying a technique from compositional data analysis, the additive log-ratio transformation [26], absolute abundances are replaced with the logarithms of pairwise ratios of the abundances. This transformation turns gLV models into cLV models, whose variables represent relative abundances.

To derive the cLV equations, let us denote the sum of all species abundances by $N=\sum_{i=1}^{n}x_{i}$, and the relative abundance of each species by $\pi_{i}=\frac{x_{i}}{N}$. Note that both *N* and $\pi_{i}$ are time-dependent. Then, by setting the mean community size to 1, the following equation is obtained:

(8) $$\frac{d}{dt}\log\left(\frac{\pi_{i}}{\pi_{n}}\right)={\bar{g}}_{i}+\sum_{j=1}^{n}{\bar{A}}_{ij}\cdot \pi_{j}\left(t\right),$$

where ${\bar{A}}_{ij}=A_{ij}-A_{nj}$. It is possible to include an additional term in (8) to account for external disturbances:

(9) $$\frac{d}{dt}\log\left(\frac{\pi_{i}}{\pi_{n}}\right)={\bar{g}}_{i}+\sum_{j=1}^{n}{\bar{A}}_{ij}\cdot \pi_{j}\left(t\right)+\sum_{p=1}^{n_{u}}{\bar{B}}_{ip}\cdot u_{p}=:F_{i}.$$

Equation (9) must be rewritten as (1) in order to perform SIO analysis, which leads to

(10) $$\frac{d}{dt}\pi_{i}=\pi_{i}\cdot \left(F_{i}-\bar{F}\right),$$

with $\bar{F}=\sum_{j=1}^{n-1}\pi_{j}\cdot F_{j}.$

### 3.2. Species–Metabolite Interaction Models (SMI)

It has been argued that the emergent dynamics of certain microbial communities are more faithfully represented as competition among species for a common resource, instead of the predator–prey relationship implied by SSI models [15]. Since the resources are typically metabolites, the resulting models are called species–metabolite interaction models or SMI. They have the following general structure:

(11) $$\begin{array}{ccc} {\dot{x}}_{i} & = & x_{i}\sum_{j=1}^{n_{m}}f_{j}^{i}\left(x_{i},m_{j}\right),\\ {\dot{m}}_{j} & = & \sum_{i=1}^{n}\sum_{l=1}^{n_{m}}h_{il}^{j}\left(x_{i},m_{l}\right), \end{array}$$

where each of the $n_{m}$ metabolites is denoted by $m_{j}$. Several choices of interaction functions $f_{j}^{i}$ and $h_{il}^{j}$ are possible.

#### 3.2.1. Quadratic Species–Metabolite Interaction Models (QSMI)

The simplest SMI models assume quadratic terms. Ref. [15] argued that this assumption yields a faithful representation of a well-mixed system, and included constant dilution terms for metabolites ($d_{i}$) and microorganisms ($d_{j}^{*}$). The resulting model is of the form

(12) $$\begin{array}{c} {\dot{x}}_{i}=x_{i}\cdot \left(\sum_{j=1}^{n_{m}}\psi_{ij}\cdot m_{j}-d_{i}\right),\\ {\dot{m}}_{j}=m_{j}\cdot \left(-\sum_{i=1}^{n}k_{ij}\cdot x_{i}-d_{j}^{*}\right)+f_{j}+\sum_{i=1}^{n}\sum_{l=1}^{n_{m}}\phi_{il}^{j}\cdot x_{i}\cdot m_{l}, \end{array}$$

where the $f_{j}$ terms allow for a constant influx of metabolite *j* and $\phi_{il}^{j}$ for its production as a by-product of a reaction involving another metabolite. We refer to the model described in (12) as QSMI, a quadratic species–metabolite interaction.

#### 3.2.2. SMI Models with Simple Monod Growth Kinetics (MSMI)

The incorporation of the Monod growth terms leads to

(13) $$\begin{array}{cc} {\dot{x}}_{i} & =x_{i}\cdot \left(\sum_{j=1}^{n_{m}}\frac{V_{ij}\cdot m_{j}}{K_{ij}+m_{j}\cdot x_{i}}-d_{i}\right),\\ {\dot{m}}_{j} & =m_{j}\cdot \left(-\sum_{i=1}^{n}\frac{V_{ij}^{*}\cdot x_{i}}{K_{ij}^{*}+m_{j}\cdot x_{i}}-d_{j}^{*}\right)+f_{j}+\sum_{i=1}^{n}\sum_{l=1}^{n_{m}}\frac{\phi_{il}^{j}\cdot x_{i}\cdot m_{l}}{K_{il}^{*}+x_{i}\cdot m_{l}}, \end{array}$$

We refer to model (13) as MSMI. The parameterization resulting from Monod growth kinetics has more parameters than the QSMI one. A simplified version of this class of models (including only one metabolite) appeared in [16], where a consumer–resource model using Monod kinetics was proposed as a means to explain the diversity of microbial communities having different growth rates.

### 3.3. A Phage Cocktail Model (PC)

A particular type of microbial community includes bacteriophage viruses, also called phages. Thanks to their ability to infect specific bacteria, phages provide an alternative to antibiotics in therapeutic applications. Many context-specific models involving phages have been proposed, including some [27,28] that describe their role in antimicrobial resistance. Here, we consider an illustrative case study: a phage cocktail model presented by Li et al. [29] that includes two bacterial strains, one sensitive (*S*) and one resistant (*R*) to therapy, the two phages that target them ($P_{S}$ and $P_{R}$, respectively), and the immune response I.

(14) $$\begin{array}{cc} \dot{S} & =ri\cdot S\cdot \left(1-\frac{S+R}{K_{C}}\right)\cdot \left(1-\mu \right)-S\cdot F\left(P_{S}\right)-\frac{\epsilon \cdot I\cdot S}{1+\left(S+R\right)/K_{D}},\\ \dot{R} & =\left(r^{\prime }\cdot R+\mu \cdot r\cdot S\right)\cdot \left(1-\frac{S+R}{K_{C}}\right)-R\cdot F\left(P_{R}\right)-\frac{\epsilon \cdot I\cdot R}{1+\left(S+R\right)/K_{D}},\\ {\dot{P}}_{S} & =\beta \cdot S\cdot F\left(P_{S}\right)-\phi \cdot S\cdot P_{S}-\omega \cdot P_{S}+\rho_{S},\\ \dot{I} & =\alpha \cdot I\cdot \left(1-I/K_{I}\right)\cdot \left(\frac{S+R}{S+R+K_{N}}\right),\\ {\dot{P}}_{R} & =\beta \cdot R\cdot F\left(P_{R}\right)-\phi \cdot R\cdot P_{R}-\omega \cdot P_{R}+\rho_{R}. \end{array}$$

where the phage–bacteria interactions are given by $F\left(P_{i}\right)=\frac{\phi \cdot P_{i}}{1+P_{i}/P_{C}}$ for i∈{R,S}. Both bacterial strains are killed by the immune response, as well as by the corresponding phage; the kinetics of the immune killing is parameterized by ϵ and $K_{D}$. Both bacteria undergo logistic growth with a maximum capacity given by $K_{C}$, and sensitive bacteria may mutate into phage-resistant bacteria with a mutation probability μ. The immune response is activated by the sum of both bacterial abundances, with activation rate α and saturation given by $K_{I}.$ As for the phages, it is assumed that they have the same adsorption rate ϕ, burst size β, and decay rate ω, but different injection rates, $\rho_{S}$ and $\rho_{R}$. The former three parameters are considered unknown, but the injection rates are assumed known since we are modelling a phage therapy application.

Li et al. [29] also presented a scaled version of (14), which has a state vector given by $x=\left[x_{1},x_{2},x_{3},x_{4},x_{5}\right]=\left[\frac{S}{K_{D}},\frac{R}{K_{D}},\frac{P_{S}}{P_{C}},\frac{I}{K_{I}},\frac{P_{R}}{P_{C}}\right]$ and the following equations:

(15) $$\begin{array}{ccc} {\dot{x}}_{1} & = & r\cdot x_{1}\cdot \left(1-\frac{x_{1}+x_{2}}{K_{CD}}\right)\cdot \left(1-\mu \right)-K_{PD}\cdot x_{1}\cdot I\left(x_{3}\right)-\frac{\tilde{\epsilon }\cdot x_{4}\cdot x_{1}}{1+x_{1}+x_{2}},\\ {\dot{x}}_{2} & = & \left(r^{\prime }\cdot x_{2}+r\cdot x_{1}\right)\cdot \left(1-\frac{x_{1}+x_{2}}{K_{CD}}\right)\cdot \mu -K_{PD}\cdot x_{2}\cdot I\left(x_{5}\right)-\frac{\tilde{\epsilon }\cdot x_{4}\cdot x_{2}}{1+x_{1}+x_{2}},\\ {\dot{x}}_{3} & = & \beta \cdot x_{1}\cdot I\left(x_{3}\right)-\psi \cdot x_{1}\cdot x_{3}-\omega \cdot x_{3}+q\cdot u_{1},\\ {\dot{x}}_{4} & = & \alpha \cdot x_{4}\cdot \left(1-x_{4}\right)\cdot \left(\frac{x_{1}+x_{2}}{x_{1}+x_{2}+K_{ND}}\right),\\ {\dot{x}}_{5} & = & \beta \cdot x_{2}\cdot I\left(x_{5}\right)-\psi \cdot x_{2}\cdot x_{5}-\omega \cdot x_{5}3+q\cdot u_{2}. \end{array}$$

where $I\left(x_{i}\right)=\frac{\psi \cdot x_{i}}{1+x_{i}}$ for i∈{3,5}.

## 4. Results

We have used the approach described in Section 2 to analyse the SIO of the models described in Section 3. Since SIO depends on the type of measurements available—i.e., on which state variables can be measured—for each model structure, we consider several possible measurement configurations. Furthermore, we consider models of different dimensions (e.g., with two or three bacterial strains, and with one or two metabolites) in order to find general identifiability patterns, i.e., which parameters are always unidentifiable irrespective of the number of species included in the model. Results of the analyses are given in Table 1, Table 2, Table 3, Table 4 and Table 5. The main findings and trends are discussed in the remainder of this section.

An overview of the results of gLV models can be seen in Table 1. The gLV models are FISPO if and only if all their state variables are outputs, i.e., if their absolute abundances are measured. If only some of its state variables are measured, some interaction rates (β) are unidentifiable—specifically, the ones related to the interactions among the unmeasured species and the other species. The interaction rates between two measured species remain identifiable. In contrast, when the measurements consist of relative abundances, all the interaction rates (β) are unidentifiable. Growth rates (*r*) are always identifiable, both with absolute and relative measurements. As for observability, a state variable is observable if and only if it is measured in absolute terms.

Table 2 shows the results of the cLV models. Their dimensions have been reduced taking into account that $x_{n}$ = 1 −$\sum_{i=1}^{n-1}x_{i}$. These models were introduced for the purpose of achieving observability, a goal that they achieve for all measurement configurations. Their identifiability is strongly influenced by the existence of (measured) inputs. In the absence of inputs, all the model parameters are unidentifiable. When we introduce an input, the parameters related to the external perturbations (*B*) become identifiable, regardless of which variables are measured.

Table 3 and Table 4 summarize the results of the SMI models. Despite their different kinetics, all of their state variables are observable if and only if they are outputs. As for their identifiability, all the dilution terms *d* and $d^{*}$ are always identifiable. The constant flux of a metabolite (*f*) is identifiable only if the abundance of that metabolite is an output. The identifiability of other parameters depends on the type of kinetics. In QSMI models, *k* terms are always unidentifiable. If $m_{r}$ is an output, $\psi_{*r}$ (where the * means all the possible indexes) are identifiable. When we measure more than one metabolite, some ϕ become identifiable: if $x_{i}$ is observed, $\phi_{i1}^{2}$ and $\phi_{i2}^{1}$ are identifiable. In MSMI models, $V^{*}$ are all always unidentifiable. If $x_{i}$ is an output, $V_{i*}$ are identifiable. If $m_{r}$ is observed, $\phi_{*s}^{r}$ are identifiable. Finally, if $x_{i}$ and $m_{r}$ are both outputs, $K_{ir}$ and $K_{ir}^{*}$ are identifiable.

Lastly, the original formulation of the PC model is FISPO if and only if the immune response (*I*) is observed (Table 5). If *I* is not measured, it becomes unobservable, even if the other variables are outputs. In this case, measuring either bacterial abundances (S,R) or phages ($P_{S},P_{R}$) yields identical results; measuring both sets does not improve the identifiability nor observability with respect to measuring a single one. For its scaled version, of all the possible output combinations, we have considered those that are experimentally more feasible: ($x_{1}$, $x_{2}$, $x_{3}$, $x_{4}$, $x_{5}$) ($x_{1}$, $x_{2}$, $x_{3}$, $x_{5}$) ($x_{1}$, $x_{2}$, $x_{4}$) ($x_{1}$, $x_{3}$, $x_{4}$) ($x_{2}$, $x_{4}$, $x_{5}$) ($x_{3}$, $x_{4}$, $x_{5}$) ($x_{1}$, $x_{2}$) ($x_{1}$, $x_{4}$) ($x_{2}$, $x_{4}$) ($x_{3}$, $x_{5}$) ($x_{4}$). In all these cases, the model is FISPO.

## 5. Discussion and Conclusions

We have analysed the structural identifiability and observability (SIO) of more than a hundred variants of the most common microbial community models. For each type of model structure, we considered different dimensions, in order to obtain general results, and different output configurations, so as to establish which measurements are more useful for identification purposes.

Notable examples of the insights obtained in this study include the finding that, for generalized Lotka–Volterra models (gLV), all the microbial species present in the community must be measured in order to achieve full observability and identifiability. In contrast, if one is only interested in determining their growth rates, it is sufficient to measure a single species. Another species–species interaction formalism, the cLV, has better observability properties than gLV at the expense of worse identifiability. As for species–metabolite interaction models, an interesting conclusion is that the only observable state variables are the measured ones, for both QSMI and MSMI model classes. Finally, the SIO of the phage cocktail model that we have analysed exhibits a strong dependency on the possibility of measuring the immune response. A scaled version of this model, which has reduced dimensions, achieves full SIO at the cost of losing the biological meaning of its reparameterized variables.

Thus, our results have revealed the limitations of several model variants, and facilitate the tasks of choosing a model that is appropriate for a particular application and designing the corresponding experimental setup. While it would be impossible to analyse all possible model structures that could be used for the study of microbial communities in a short paper, the present work has studied the most relevant ones, and serves as a demonstration of the methodology that can be applied for other models.

## Figures and Tables

**Table 1:** **gLV.** Results for the generalized Lotka–Volterra models.

x	y	Id	Non-Id	Obs	Non-Obs
$x_{1}$, $x_{2}$	$x_{1}$, $x_{2}$	all	-	all	-
	$x_{i}$	$r_{*}$, $\beta_{*i}$	$\beta_{*j}$	$x_{i}$	$x_{j}$
	RM	$r_{*}$	$\beta_{*}$	-	$x_{*}$
$x_{1}$, $x_{2}$, $x_{3}$	$x_{1}$, $x_{2}$, $x_{3}$	all	-	all	-
	$x_{j}$, $x_{k}$	$r_{*}$, $\beta_{*j}$, $\beta_{*k}$	$\beta_{*i}$	$x_{j}$, $x_{k}$	$x_{i}$
	$x_{i}$	$r_{*}$, $\beta_{*i}$	$\beta_{*j}$, $\beta_{*k}$	$x_{i}$	$x_{j}$, $x_{k}$
	RM	$r_{*}$	$\beta_{*}$	-	$x_{*}$

An asterisk as a subscript (${}_{*}$) refers to the set of all the possible subindexes. We use subscripts *i*, *j*, *k* such that i≠j, i≠k, j≠k. RM (relative measures) means all the possible combinations of the form $\frac{xi}{\sum_{j=1}^{n}x_{j}}$.

**Table 2:** **cLV.** Results for the composite Lotka–Volterra models.

x	u	y	Id	Non-Id	Obs	Non-Obs
$\pi_{1}$	-	$\pi_{1}$	-	all	all	-
	$u_{1}$	$\pi_{1}$	$B_{*}$	$g_{*}$, $A_{*}$	all	-
$\pi_{1}$, $\pi_{2}$	-	$\pi_{1}$, $\pi_{2}$ $\pi_{i}$	-	all	all	-
	$u_{1}$	$\pi_{1}$, $\pi_{2}$ $\pi_{i}$	$B_{*}$	$g_{*}$, $A_{*}$	all	-

An asterisk as a subscript (${}_{*}$) refers to the set of all the possible subindexes.

**Table 3:** **QSMI.** Results for the quadratic species–metabolite interaction models.

x	y	Id	Non-Id	Obs	Non-Obs
$x_{1}$, $x_{2}$, $m_{1}$	$x_{*}$, $m_{1}$	$d_{*}$, $d_{*}^{*}$, $f_{*}$, $\psi_{*}$	$k_{*}$, $\phi_{*}$	all	-
	$x_{i}$, $m_{1}$	$d_{*}$, $d_{*}^{*}$, $f_{*}$, $\psi_{*}$	$k_{*}$, $\phi_{*}$	$x_{i}$, $m_{1}$	$x_{j}$
	$m_{1}$	$d_{*}$, $d_{*}^{*}$, $f_{*}$, $\psi_{*}$	$k_{*}$, $\phi_{*}$	$m_{1}$	$x_{1}$, $x_{2}$
	$x_{*}$	$d_{*}$, $d_{*}^{*}$	$k_{*}$, $\phi_{*}$, $f_{*}$, $\psi_{*}$	$x_{*}$	$m_{*}$
	$x_{i}$	$d_{*}$, $d_{*}^{*}$	$k_{*}$, $\phi_{*}$, $f_{*}$, $\psi_{*}$	$x_{i}$	$x_{j}$, $m_{1}$
$x_{1}$, $x_{2}$, $x_{3}$, $m_{1}$	$x_{*}$, $m_{1}$	$d_{*}$, $d_{*}^{*}$, $f_{*}$, $\psi_{*}$	$k_{*}$, $\phi_{*}$	all	-
	$x_{j}$, $x_{k}$, $m_{1}$	$d_{*}$, $d_{*}^{*}$, $f_{*}$, $\psi_{*}$	$k_{*}$, $\phi_{*}$	$x_{j}$, $x_{k}$, $m_{1}$	$x_{i}$
	$x_{i}$, $m_{1}$	$d_{*}$, $d_{*}^{*}$, $f_{*}$, $\psi_{*}$	$k_{*}$, $\phi_{*}$	$x_{i}$, $m_{1}$	$x_{j}$, $x_{k}$
	$m_{1}$	$d_{*}$, $d_{*}^{*}$, $f_{*}$, $\psi_{*}$	$k_{*}$, $\phi_{*}$	$m_{1}$	$x_{1}$, $x_{2}$, $x_{3}$
	$x_{*}$	$d_{*}$, $d_{*}^{*}$	$k_{*}$, $\phi_{*}$, $f_{*}$, $\psi_{*}$	$x_{*}$	$m_{*}$
	$x_{j}$, $x_{k}$	$d_{*}$, $d_{*}^{*}$	$k_{*}$, $\phi_{*}$, $f_{*}$, $\psi_{*}$	$x_{j}$, $x_{k}$	$x_{i}$, $m_{1}$
	$x_{i}$	$d_{*}$, $d_{*}^{*}$	$k_{*}$, $\phi_{*}$, $f_{*}$, $\psi_{*}$	$x_{i}$	$x_{j}$, $x_{k}$, $m_{i}$
$x_{1}$, $x_{2}$, $m_{1}$, $m_{2}$	$x_{*}$, $m_{*}$	$d_{*}$, $d_{*}^{*}$, $f_{*}$, $\psi_{*}$, $\phi_{*1}^{2}$, $\phi_{*2}^{1}$	$k_{*}$, $\phi_{*1}^{1}$, $\phi_{*2}^{2}$	all	-
	$x_{i}$, $m_{*}$	$d_{*}$, $d_{*}^{*}$, $f_{*}$, $\psi_{*}$, $\phi_{i1}^{2}$, $\phi_{i2}^{1}$	$k_{*}$, $\phi_{i1}^{1}$, $\phi_{i2}^{2}$, $\phi_{j**}$	$x_{i}$, $m_{*}$	$x_{j}$
	$m_{*}$	$d_{*}$, $d_{*}^{*}$, $f_{*}$, $\psi_{*}$	$k_{*}$, $\phi_{*}$	$m_{*}$	$x_{*}$
	$x_{*}$, $m_{r}$	$d_{*}$, $d_{*}^{*}$, $f_{r}$, $\psi_{*r}$,	$k_{*}$, $\phi_{*}$, $f_{s}$, $\psi_{*s}$	$x_{*}$, $m_{r}$	$m_{s}$
	$x_{i}$, $m_{r}$	$d_{*}$, $d_{*}^{*}$, $f_{r}$, $\psi_{*r}$	$k_{*}$, $\phi_{*}$, $f_{s}$, $\psi_{*s}$,	$x_{i}$, $m_{r}$	$x_{j}$, $m_{s}$
	$m_{r}$	$d_{*}$, $d_{*}^{*}$, $f_{r}$, $\psi_{*r}$	$k_{*}$, $\phi_{*}$, $f_{s}$, $\psi_{*s}$,	$m_{r}$	$x_{*}$, $m_{s}$
	$x_{*}$	$d_{*}$, $d_{*}^{*}$	$k_{*}$, $f_{*}$, $\psi_{*}$, $\phi_{*}$	$x_{*}$	$m_{*}$
	$x_{i}$	$d_{*}$, $d_{*}^{*}$	$k_{*}$, $f_{*}$, $\psi_{*}$, $\phi_{*}$	$x_{i}$	$x_{j}$, $m_{*}$
$x_{1}$, $x_{2}$, $x_{3}$, $m_{1}$, $m_{2}$	$x_{*}$, $m_{*}$	$d_{*}$, $d_{*}^{*}$, $f_{*}$, $\psi_{*}$, $\phi_{*1}^{2}$, $\phi_{*2}^{1}$	$k_{*}$, $\phi_{*1}^{1}$, $\phi_{*2}^{2}$	all	-
	$x_{j}$, $x_{l}$, $m_{*}$	$d_{*}$, $d_{*}^{*}$, $f_{*}$, $\psi_{*}$, $\phi_{j1}^{2}$, $\phi_{j2}^{1}$, $\phi_{l1}^{2}$, $\phi_{l2}^{1}$	$k_{*}$, $\phi_{j1}^{1}$, $\phi_{j2}^{2}$, $\phi_{l1}^{1}$, $\phi_{l2}^{2}$, $\phi_{3*}^{*}$	$x_{j}$, $x_{l}$, $m_{*}$	$x_{i}$
	$x_{i}$, $m_{*}$	$d_{*}$, $d_{*}^{*}$, $f_{*}$, $\psi_{*}$, $\phi_{i1}^{2}$, $\phi_{i2}^{1}$	$k_{*}$, $\phi_{i1}^{1}$, $\phi_{i2}^{2}$, $\phi_{j*}^{*}$, $\phi_{l*}^{*}$	$x_{i}$, $m_{*}$	$x_{j}$, $x_{l}$
	$m_{*}$	$d_{*}$, $d_{*}^{*}$, $f_{*}$, $\psi_{*}$	$k_{*}$, $\phi_{*}$	$m_{*}$	$x_{*}$
	$x_{*}$, $m_{r}$	$d_{*}$, $d_{*}^{*}$, $f_{r}$, $\psi_{*r}$	$k_{*}$, $\phi_{*}$, $f_{s}$, $\psi_{*s}$	$x_{*}$, $m_{r}$	$m_{s}$
	$x_{j}$, $x_{l}$, $m_{r}$	$d_{*}$, $d_{*}^{*}$, $f_{r}$, $\psi_{*r}$	$k_{*}$, $\phi_{*}$, $f_{s}$, $\psi_{*s}$	$x_{j}$, $x_{l}$, $m_{r}$	$x_{i}$, $m_{s}$
	$x_{i}$, $m_{r}$	$d_{*}$, $d_{*}^{*}$, $f_{r}$, $\psi_{*r}$	$k_{*}$, $\phi_{*}$, $f_{s}$, $\psi_{*s}$	$x_{i}$, $m_{r}$	$x_{j}$, $x_{l}$, $m_{s}$
	$m_{r}$	$d_{*}$, $d_{*}^{*}$, $f_{r}$, $\psi_{*r}$	$k_{*}$, $\phi_{*}$, $f_{s}$, $\psi_{*s}$	$m_{r}$	$x_{*}$, $m_{s}$
	$x_{*}$	$d_{*}$, $d_{*}^{*}$	$k_{*}$, $f_{*}$, $\psi_{*}$, $\phi_{*}$	$x_{*}$	$m_{*}$
	$x_{j}$, $x_{l}$	$d_{*}$, $d_{*}^{*}$	$k_{*}$, $f_{*}$, $\psi_{*}$, $\phi_{*}$	$x_{j}$, $x_{l}$	$x_{i}$, $m_{*}$
	$x_{i}$	$d_{*}$, $d_{*}^{*}$	$k_{*}$, $f_{*}$, $\psi_{*}$, $\phi_{*}$	$x_{i}$	$x_{j}$, $x_{l}$, $m_{*}$

An asterisk as a subscript (${}_{*}$) refers to the set of all the possible subindexes. We use subscripts *i*, *j*, *l* such that i≠j, i≠l, j≠l, and *r*, *s* such that *r* ≠ *s*.

**Table 4:** **MSMI.** Results for the species–metabolite interaction models with Monod growth.

x	y	Id	Non-Id	Obs	Non-Obs
$x_{1}$, $x_{2}$, $m_{1}$	$x_{*}$, $m_{1}$	$d_{*}$, $d_{1}^{*}$, $V_{*}$, $K_{*}$, $K_{*}^{*}$, $f_{1}$	$V_{*}^{*}$, $\phi_{*}$	all	-
	$x_{i}$, $m_{1}$	$d_{*}$, $d_{1}^{*}$, $V_{i1}$, $K_{i1}$, $K_{i1}^{*}$, $f_{1}$	$V_{j1}$, $K_{j1}$, $V_{*}^{*}$, $K_{j1}^{*}$, $\phi_{*}$	$x_{i}$, $m_{1}$	$x_{j}$
	$m_{1}$	$d_{*}$, $d_{1}^{*}$, $f_{1}$	$V_{*}$, $K_{*}$, $V_{*}^{*}$, $K_{*}^{*}$, $\phi_{*}$	$m_{1}$	$x_{1}$, $x_{2}$
	$x_{*}$	$d_{*}$, $d_{1}^{*}$, $V_{*}$	$K_{*}$, $V_{*}^{*}$, $K_{*}^{*}$, $f_{1}$, $\phi_{*}$	$x_{*}$	$m_{*}$
	$x_{i}$	$d_{*}$, $d_{*}^{*}$, $V_{i1}$	$V_{j1}$, $K_{*}$, $V_{*}^{*}$, $K_{*}^{*}$, f1, $\phi_{*}$	$x_{i}$	$x_{j}$, $m_{1}$
$x_{1}$, $x_{2}$, $m_{1}$, $m_{2}$	$x_{*}$, $m_{*}$	$d_{*}$, $d_{*}^{*}$, $V_{*}$, $K_{*}$, $K_{*}^{*}$, $f_{*}$, $\phi_{*2}^{1}$, $\phi_{*1}^{2}$	$V_{*}^{*}$, $\phi_{*1}^{1}$, $\phi_{*2}^{2}$	all	-
	$x_{i}$, $m_{*}$	$d_{*}$, $d_{*}^{*}$, $V_{i*}$, $K_{i*}$, $K_{i*}^{*}$, $f_{*}$, $\phi_{*2}^{1}$, $\phi_{*1}^{2}$	$V_{j*}$, $K_{j*}$, $V_{*}^{*}$, $K_{j*}^{*}$, $\phi_{*1}^{1}$, $\phi_{*2}^{2}$	$x_{i}$, $m_{*}$	$x_{j}$
	$m_{*}$	$d_{*}$, $d_{*}^{*}$, $f_{*}$, $\phi_{*2}^{1}$, $\phi_{*1}^{2}$	$V_{*}$, $K_{*}$, $V_{*}^{*}$, $K_{*}^{*}$, $\phi_{*1}^{1}$, $\phi_{*2}^{2}$	$m_{*}$	$x_{*}$
	$x_{*}$, $m_{r}$	$d_{*}$, $d_{*}^{*}$, $V_{*}$, $K_{*r}$, $K_{*r}^{*}$, $f_{r}$, $\phi_{*s}^{r}$	$K_{*s}$, $V_{*}^{*}$, $K_{*s}^{*}$, $f_{s}$, $\phi_{*r}^{s}$, $\phi_{*r}^{r}$, $\phi_{*s}^{s}$	$x_{*}$, $m_{r}$	$m_{s}$
	$x_{i}$, $m_{r}$	$d_{*}$, $d_{*}^{*}$, $V_{i*}$, $K_{ir}$, $K_{ir}^{*}$, $f_{r}$, $\phi_{*s}^{r}$	$V_{j*}$, $K_{is}$, $K_{jr}$, $K_{js}$, $V_{*}^{*}$, $K_{is}^{*}$, $K_{jr}^{*}$, $K_{js}^{*}$, $f_{s}$, $\phi_{*r}^{s}$, $\phi_{*r}^{r}$, $\phi_{*s}^{s}$	$x_{i}$, $m_{r}$	$x_{j}$, $m_{s}$
	$m_{r}$	$d_{*}$, $d_{*}^{*}$, $f_{r}$, $\phi_{*s}^{r}$	$V_{*}$, $K_{*}$, $V_{*}^{*}$, $K_{*}^{*}$, $f_{s}$, $\phi_{*r}^{s}$, $\phi_{*r}^{r}$, $\phi_{*s}^{s}$	$m_{r}$	$x_{*}$, $m_{s}$
	$x_{*}$	$d_{*}$, $d_{*}^{*}$, $V_{*}$	$K_{*}$, $V_{*}^{*}$, $K_{*}^{*}$, $f_{*}$, $\phi_{*}$	$x_{*}$	$m_{*}$
	$x_{i}$	$d_{*}$, $d_{*}^{*}$, $V_{i*}$	$V_{j*}$, $K_{*}$, $V_{*}^{*}$, $K_{*}^{*}$, $f_{*}$, $\phi_{*}$	$x_{i}$	$x_{j}$, $m_{*}$
$x_{1}$, $x_{2}$, $x_{3}$, $m_{1}$, $m_{2}$	$x_{*}$, $m_{*}$	$d_{*}$, $d_{*}^{*}$, $V_{*}$, $K_{*}$, $K_{*}^{*}$, $f_{*}$, $\phi_{*2}^{1}$, $\phi_{*1}^{2}$	$V_{*}^{*}$, $\phi_{*1}^{1}$, $\phi_{*2}^{2}$	all	-
	$x_{j}$, $x_{l}$, $m_{*}$	$d_{*}$, $d_{*}^{*}$, $V_{j*}$, $V_{l*}$, $K_{j*}$, $K_{l*}$, $K_{j*}^{*}$, $K_{l*}^{*}$, $f_{*}$, $\phi_{*2}^{1}$, $\phi_{*1}^{2}$	$V_{i*}$, $K_{i*}$, $V_{*}^{*}$, $K_{i*}^{*}$, $\phi_{*1}^{1}$, $\phi_{*2}^{2}$	$x_{j}$, $x_{l}$, $m_{*}$	$x_{i}$
	$x_{i}$, $m_{*}$	$d_{*}$, $d_{*}^{*}$, $V_{i*}$, $K_{i*}$, $K_{i*}^{*}$, $f_{*}$, $\phi_{*2}^{1}$, $\phi_{*1}^{2}$	$V_{j*}$, $V_{l*}$, $K_{j*}$, $K_{l*}$, $V_{*}^{*}$, $K_{j*}^{*}$, $K_{l*}^{*}$, $\phi_{*1}^{1}$, $\phi_{*2}^{2}$	$x_{i}$, $m_{*}$	$x_{j}$, $x_{l}$
	$m_{*}$	$d_{*}$, $d_{*}^{*}$, $f_{*}$, $\phi_{*2}^{1}$, $\phi_{*1}^{2}$	$V_{*}$, $K_{*}$, $V_{*}^{*}$, $K_{*}^{*}$, $\phi_{*1}^{1}$, $\phi_{*2}^{2}$	$m_{*}$	$x_{*}$
	$x_{*}$, $m_{r}$	$d_{*}$, $d_{*}^{*}$, $V_{*}$, $K_{*r}$, $K_{*r}^{*}$, $f_{r}$, $\phi_{*s}^{r}$	$K_{*s}$, $V_{*}^{*}$, $K_{*s}^{*}$, $f_{s}$, $\phi_{*r}^{s}$, $\phi_{*r}^{r}$, $\phi_{*s}^{s}$	$x_{*}$, $m_{r}$	$m_{s}$
	$x_{j}$, $x_{l}$, $m_{r}$	$d^{*}$, $d_{*}^{*}$, $V_{j*}$, $V_{l*}$, $K_{jr}$, $K_{lr}$, $K_{jr}^{*}$, $K_{lr}^{*}$, $f_{r}$, $\phi_{*s}^{r}$	$V_{i*}$, $K_{js}$, $K_{ls}$, $K_{i*}$, $V_{*}^{*}$, $K_{js}^{*}$, $K_{ls}^{*}$, $K_{i*}^{*}$, $f_{s}$, $\phi_{*r}^{s}$, $\phi_{*r}^{r}$, $\phi_{*s}^{s}$	$x_{j}$, $x_{l}$, $m_{r}$	$x_{i}$, $m_{s}$
	$x_{i}$, $m_{r}$	$d_{*}$, $d_{*}^{*}$, $V_{i*}$, $K_{ir}$, $K_{ir}^{*}$, $f_{r}$, $\phi_{*s}^{r}$	$V_{j*}$, $V_{l*}$, $K_{is}$, $K_{j*}$, $K_{l*}$, $V_{*}^{*}$, $K_{is}^{*}$, $K_{j*}^{*}$, $K_{l*}^{*}$, $f_{s}$, $\phi_{*r}^{s}$, $\phi_{*r}^{r}$, $\phi_{*s}^{s}$	$x_{i}$, $m_{r}$	$x_{j}$, $x_{l}$, $m_{s}$
	$m_{r}$	$d_{*}$, $d_{*}^{*}$, $f_{r}$, $\phi_{*s}^{r}$	$V_{*}$, $K_{*}$, $V_{*}^{*}$, $K_{*}^{*}$, $f_{s}$, $\phi_{*r}^{s}$, $\phi_{*r}^{r}$, $\phi_{*s}^{s}$	$m_{r}$	$x_{*}$, $m_{s}$
	$x_{*}$	$d_{*}$, $d_{*}^{*}$, $V_{*}$	$K_{*}$, $V_{*}^{*}$, $K_{*}^{*}$, $f_{*}$, $\phi_{*}$	$x_{*}$	$m_{*}$
	$x_{j}$, $x_{l}$	$d^{*}$, $d_{*}^{*}$, $V_{j*}$, $V_{l*}$	$V_{i*}$, $K_{*}$, $V_{*}^{*}$, $K_{*}^{*}$, $f_{*}$, $\phi_{*}$	$x_{j}$, $x_{l}$	$x_{i}$, $m_{*}$
	$x_{i}$	$d_{*}$, $d_{*}^{*}$, $V_{i*}$	$V_{j*}$, $V_{l*}$, $K_{*}$, $V_{*}^{*}$, $K_{*}^{*}$, $f_{*}$, $\phi_{*}$	$x_{i}$	$x_{j}$, $x_{l}$, $m_{*}$

An asterisk as a subscript (${}_{*}$) refers to the set of all the possible subindexes. We use subscripts *i*, *j*, *l* such that i≠j, i≠l, j≠l, and *r*, *s* such that *r* ≠ *s*.

**Table 5:** **PC.** Results for the phage cocktail models.

x	y	Id	Non-Id	Obs	Non-Obs
*I*, *S*, *R*, $P_{S}$, $P_{R}$	*I*	all	-	all	-
	*S*, *R*, $P_{S}$, $P_{R}$	*r*, $r^{\prime }$, *m*, $P_{C}$, $K_{C}$, $K_{D}$, $K_{N}$, β, α, ϕ, ω	*E*, $K_{I}$	*S*, *R*, $P_{S}$, $P_{R}$	*I*
	*S*, *R*	*r*, $r^{\prime }$, *m*, $P_{C}$, $K_{C}$, $K_{D}$, $K_{N}$, β, α, ϕ, ω	*E*, $K_{I}$	*S*, *R*, $P_{S}$, $P_{R}$	*I*
	$P_{S}$, $P_{R}$	*r*, $r^{\prime }$, *m*, $P_{C}$, $K_{C}$, $K_{D}$, $K_{N}$, β, α, ϕ, ω	*E*, $K_{I}$	*S*, *R*, $P_{S}$, $P_{R}$	*I*
$x_{1}$, $x_{2}$, $x_{3}$, $x_{4}$, $x_{5}$	Any	all	-	all	-

‘Any’ means that all combinations listed in Section 4 have given the same result.

## Data Availability

The scripts used to obtain the results presented in this paper can be found in the ‘models’ folder of STRIKE-GOLDD, https://github.com/afvillaverde/strike-goldd accessed on 13 April 2023.

## Abbreviations

The following abbreviations are used in this manuscript:

ODE	Ordinary Differential Equation
SIO	Structural Identifiability and Observability
gLV	generalized Lotka–Volterra
SLI	Structurally Locally Identifiable
SU	Structurally Unidentifiable
SSI	Species–Species Interaction
SMI	Species–Metabolite Interaction
QSMI	Quadratic Species–Metabolite Interaction
MSMI	SMI model with Monod growth kinetics
cLV	composite Lotka–Volterra
PC	Phage Cocktail
